# Insulin receptor Arg717 and IGF-1 receptor Arg704 play a key role in ligand binding and in receptor activation

**DOI:** 10.1098/rsob.230142

**Published:** 2023-11-08

**Authors:** Anna Kertisová, Lenka Žáková, Kateřina Macháčková, Aleš Marek, Pavel Šácha, Petr Pompach, Jiří Jiráček, Irena Selicharová

**Affiliations:** ^1^ Institute of Organic Chemistry and Biochemistry, Czech Academy of Sciences, Flemingovo nám. 2, 116 10 Prague 6, Czech Republic; ^2^ Department of Genetics and Microbiology, Faculty of Science, Charles University, 128 40 Prague 2, Czech Republic; ^3^ Institute of Biotechnology, Czech Academy of Sciences, Průmyslová 595, 252 50, Vestec, Czech Republic

**Keywords:** mutagenesis *in vitro*, peptide hormone, receptor modification, receptor tyrosine kinase, structure–function

## Abstract

The insulin receptor (IR, with its isoforms IR-A and IR-B) and the insulin-like growth factor 1 receptor (IGF-1R) are related tyrosine kinase receptors. Recently, the portfolio of solved hormone–receptor structures has grown extensively thanks to advancements in cryo-electron microscopy. However, the dynamics of how these receptors transition between their inactive and active state are yet to be fully understood. The C-terminal part of the alpha subunit (*α*CT) of the receptors is indispensable for the formation of the hormone-binding site. We mutated the *α*CT residues Arg717 and His710 of IR-A and Arg704 and His697 of IGF-1R. We then measured the saturation binding curves of ligands on the mutated receptors and their ability to become activated. Mutations of Arg704 and His697 to Ala in IGF-1R decreased the binding of IGF-1. Moreover, the number of binding sites for IGF-1 on the His697 IGF-1R mutant was reduced to one-half, demonstrating the presence of two binding sites. Both mutations of Arg717 and His710 to Ala in IR-A inactivated the receptor. We have proved that Arg717 is important for the binding of insulin to its receptor, which suggests that Arg717 is a key residue for the transition to the active conformation.

## Graphical abstract

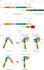

## 1. Introduction

**Figure 1.**
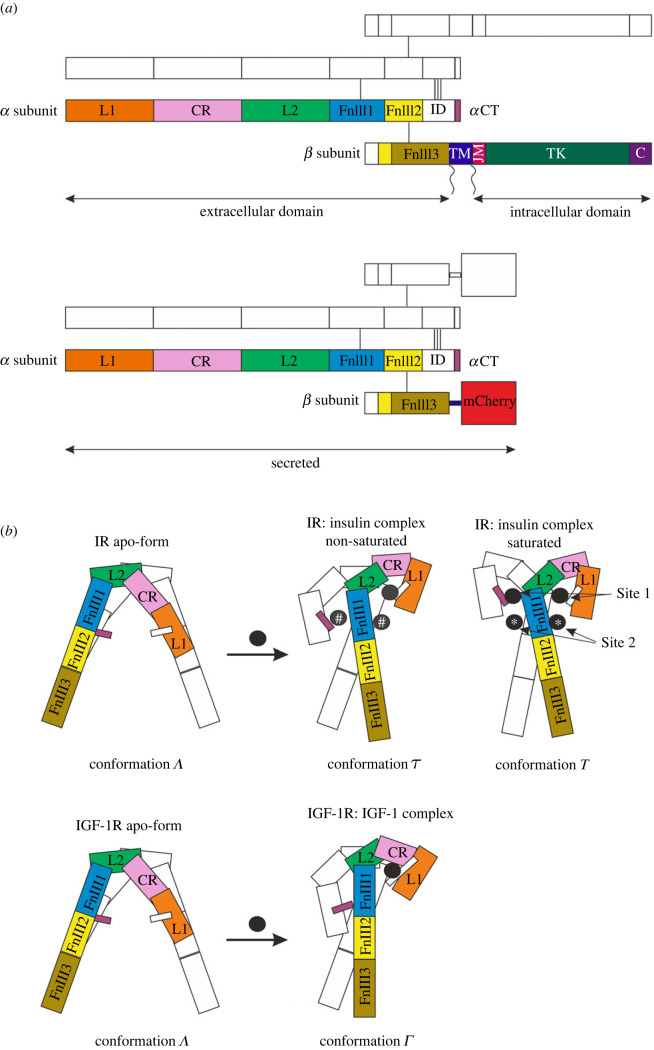
Schematic organization of IR and IGF-1R domains. (*a*) Structural domains of the full-length receptor dimer and its ectodomains conjugated with mCherry prepared in this study. Domains are marked on one half of the dimer, and the second part is sketched. The domains are named as follows: L, leucine-rich; CR, cysteine-rich; FnIII, fibronectin type III; ID, insert; TM, transmembrane; JM, juxtamembrane; TK, tyrosine kinase; C, C-terminal. Subunits are disulfide-linked, as indicated by connecting lines. For a review, see [4]. (*b*) Models of receptor ectodomain conformations in apo-form and with bound ligands. Receptor domains are named as in (*a*) and marked on one half of the dimer; the second part is sketched. The disulfides and insert domains, except the *α*CTs forming binding sites, are omitted. Circles represent ligands. The non-saturated IR complex can accommodate 1, 2 or 3 insulin molecules; the presence of ligands marked with # is optional. The conformations with a different number of insulins can differ slightly. The saturated complex with the insulin receptor has two insulins bound at Site 1 and two insulins at Site 2, marked with an asterisk. The cartoons were created based on the structures published in [4–7].

The insulin receptor (IR, with its isoforms IR-A and IR-B) and the IGF-1 receptor (IGF-1R) are related tyrosine kinase receptors mediating the signal transduction and activity of insulin and insulin-like growth factors (IGF-1 and IGF-2), which are hormones indispensable for life. In brief, insulin controls the metabolism whereas IGFs stimulate growth and development. Any malfunction of these hormones results in serious health problems such as diabetes mellitus, the metabolic syndrome, growth retardation, cancer and also neurodegenerative diseases [1,2]. Exactly how these hormones bind to their receptors and the mechanism of their signal transduction has been puzzling scientists for decades [3,4]. The schematic structure and domain organization of the receptors is presented in figure 1*a*. Hormones can cross-bind to the respective receptor and the receptors can even form hybrids [1]. The main enigma is that although both the hormones and their receptors share a high level of sequence and structural identity, as well as similar signalling pathways [8], each hormone still fulfils its own specialized tasks [1].

The distinct functions of the receptors originate to some extent from domain differences. For example, Nagao *et al.* [9] demonstrated that a single amino acid substitution in the juxtamembrane region results in a switch between the metabolic and the growth signalling pathway. Distinct activities can also arise from differences in signal gating [10] and also from differences in binding kinetics [11].

The binding kinetics of insulin to its receptors are complicated, as are those of IGF-1 binding to IGF-1R [11–13]. The binding is characterized by a curvilinear Scatchard plot [14], assuming a 1 :  2 stoichiometry (two binding sites for the hormone) and a cold ligand-accelerated dissociation of the tracer, which is typical for negative cooperativity. The dose-response curve for the accelerated dissociation is sigmoid in the case of IGF-1 on IGF-1R, but it is bell-shaped for insulin on the insulin receptor. The mathematical model that best explained all the binding data was constructed by Kiselyov *et al.* [11] and was based on the concept of a harmonic oscillator. The model considered two distinct binding sites (Site 1 and Site 2) located on two separate subunits of the receptor dimer. Site 1 and Site 2 on different subunits were crosslinked in the active conformation whereas the parent sites were separated. The separated sites could be occupied by insulin at high concentration in the case of the insulin receptor while this was not allowed for IGF-1R. This presumption allowed the modelling of a bell-shaped contra-sigmoid shape of the accelerated dissociation curves.

**Figure 2.**
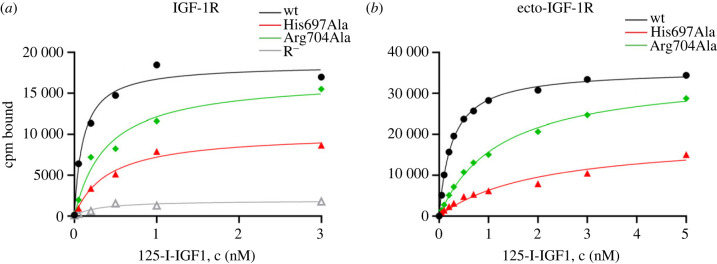
Representative saturation binding curves of IGF-1R mutants. (*a*) Whole receptors transiently expressed in R^−^ cells. (*b*) Isolated ectodomains attached to plates. ^125^I-IGF-1 is radioactively labelled human IGF-1. Individual curves including non-specific binding are presented in electronic supplementary material, figures S10–S11.

Recently published crystal and cryo-electron microscopic (cryo-EM) structures have provided details on the locations of binding sites on both receptors and brought new insights into the mechanism of how these receptors are activated (reviewed in [4,15–17]). The receptors in apo-form resembled an inverted V-shape [18] and the kinase domains were separated (figure 1*b*). Reconstitution of full-length IR into lipid nano discs [19] revealed a substantial structural rearrangement of the conformation of IR ectodomains from the inverted V-shape to a T-shape, bringing the transmembrane domains into close proximity, which is necessary for the activation of tyrosine kinase [3]. The T-shape conformation of the insulin receptor was described simultaneously by two laboratories using cryo-EM [5,20]. The T-shape conformation showed two insulins bound to Site 1 and two insulins bound to Site 2 (figure 1*b*), contrary to the original presumptions used to build the kinetics model [11,21], which presumed that the active conformation was asymmetric and that Sites 1 and 2 were simultaneously occupied by one insulin molecule. It seems that the sites cooperate during the transition to the active conformation but that they are not crosslinked in the final conformation [5,6,22]. However, the dynamics of the transitions between the inactive and the active state are still not fully understood. Non-saturated insulin–IR complexes (with 1, 2 or 3 insulins bound) have also been characterized, and all exhibit one of several asymmetric crane-like 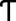-structures (figure 2*b*) [6,22,23]. It is still not clear whether these non-saturated insulin receptor structures represent transient states between the apo-form and the T-conformation, or, more probably, whether they are the true active conformations reflecting physiological conditions at low insulin concentration. It has also been speculated that these various active conformations (a T-shape versus a crane-like 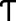-shape), which differ in the distance of potential membrane entry, might have an impact on signal transduction and be a deciding factor affecting variations in signalling output [17,22]. This is supported by the fact that the activation of the insulin receptor by insulin analogues or mimetics has been found to exhibit signalling bias. The structures of complexes between insulin analogues and the insulin receptor have been found to differ slightly from that of insulin [17]. Apparently, activation of the insulin receptor can be achieved by multiple mechanisms.

This does not apply to IGF-1R, which seems to be activated by a single ligand and adopts an asymmetric Γ-shape conformation. that is stabilized by contacts within the unliganded L1′ and FnIII-2′ domains and coupled *α*CT peptides (figure 2*b*) [7,24]. Papers describing the structures of IGF-1R complexed with two ligands have appeared only very recently. These symmetric structures seem to represent inactive forms of IGF-1R [25–27]. The Site-2 insulin-binding mode observed in IR T-shaped structures therefore most probably does not apply to the binding of IGF-1 to IGF-1R, as suggested also by mutational studies [28,29].

Although advanced methods, such as Cryo-EM, are quickly filling gaps in our understanding of IR and IGF-1R binding and activation, classical methodologies, for example involving mutational and kinetics measurements, are still greatly useful. As already mentioned, the insulin amino acid residues important for receptor binding have been mapped in phylogenetic, mutational and crosslinking studies and classified as Site 1 and Site 2 residues [3,4,30]. The deduced Site 2 binding surface of insulin encompassed the B-chain residues HisB10, GluB13 and LeuB17, and the A-chain residues Ile A10, Ser A12, Leu A13 and Glu A17. We have recently performed a thorough study mapping how mutations of the suggested Site 2 residues in the insulin A-chain (Ile A10, Ser A12, Leu A13 and Glu A17) and equivalent positions in both IGFs affect the binding of hormones to both isoforms of IR and IGF-1R [28,30]. We and others [29] have concluded that the positions corresponding to Ile A10 and Leu A13, which are important for Site 2 binding in the insulin receptor, do not play any role in IGF-1R binding. The role of the highly conserved Glu at position A17 in insulin, 58 in IGF-1 and 57 in IGF-2 in receptor binding was more complicated. The mutation of insulin GluA17 to His resulted in only 7% affinity towards IR-A. The mutation of Glu58 in IGF-1 to Asp or His decreased the affinity of the hormone towards both IGF-1R and IR-A. Strangely, the affinity of IGF-2 carrying the Glu57 to Asp mutation exhibited even greater affinity towards IGF-1R [28].

Among the IGF-1R structures published, an interaction was suggested between Glu58 IGF-1 and receptor *α*CT residue Arg704 [21], which is supposed to be part of Site 1 [28]. Among the IR structures, GluA17 has been shown in the T-shaped fully saturated IR cryo-EM structure to bind at insulin-binding Site 2 [5,20]. However, it was also positioned in close proximity of the IR Arg717 within insulin-binding Site 1 [5,20]. The IR Arg717 mutant was created during alanine scanning mutagenesis of ecto-IR, but this mutation had no effect on insulin binding [31]. This residue, either in IR (Arg717) or in IGF-1R (Arg704), was not mutated in any of the later works studying IGF-1R or the full-length insulin receptor [32,33].

Based on the presumption that Glu58 in IGF-1 might be important for IGF-1R binding through its interaction with Arg704, we performed a mutational study of the Arg residue in both the IGF-1R and IR-A full-length receptors and also in their respective ectodomains. For comparison and as a control, we also mutated His710 IR-A and His697 IGF-1R, the roles of which in ligand binding are well documented [21,34]. Our study brings new perspectives on the roles of these residues in ligand binding and receptor activation.

## 2. Results

### 2.1. Protein production

We introduced mutations of the *α*CT peptide to both IR-A and IGF-1R. We mutated Arg717 IR-A and Arg704 IGF-1R to elucidate their role in ligand binding. For comparison and as a control of our procedures, we also mutated His710 IR-A and His697 IGF-1R, the role of which in ligand binding has been well documented. Whole receptors were transiently expressed in R^−^ cells (fibroblasts with the deleted gene for mouse IGF-1R and minor expression of mouse IR-A, approximately 5 × 10^3^ receptors per cell [35]). The quantity and processing of the transiently expressed receptors was comparable in all cases (electronic supplementary material, figures S1–S2).

We produced the ectodomains of the wild-type receptors and of their mutants. The ectodomains were conjugated C-terminally with the fluorescent mCherry protein to facilitate their production and quantification. To confirm the introduction of the mutations, the receptor *α* subunits of the mutants and wild-type receptor ectodomains were excised from SDS-polyacrylamide gels and identified using mass spectrometry (MS). Sequence coverage and fragmentation spectra of peptides with mutation are presented in electronic supplementary material, figures S3–S9. Only the IR Arg717Ala mutant was not confirmed by MS/MS data. Nevertheless, similar IR Arg717Gln and IR Arg717Phe mutants were successfully detected.

### 2.2. IGF-1R mutants

We performed saturation binding experiments on R^−^ cells with the whole wild-type receptor and its mutants one day post-transfection. We considered only the transition between the unbound and the saturated state. This simplification allowed us to estimate the approximate equilibrium binding constants corresponding to the formation of a fully saturated high-affinity ligand–receptor complex. Representative binding curves are presented in figure 2*a*. The individual binding curves including non-specific binding are provided in electronic supplementary material, figure S10. We also isolated the IGF-1R ectodomains conjugated with the mCherry fluorescent protein. The concentration of receptors was estimated from the fluorescence measurements and an equal amount of the receptors was attached to plates. Representative binding curves are shown in figure 2*b*. The individual binding curves including non-specific binding are presented in electronic supplementary material, figure S11.

The binding of ^125^I-IGF-1 to R^−^ cells was negligible. The binding characteristics of the mutated receptors are presented in table 1 and complete data from all experiments are provided in electronic supplementary material, table S1. Binding constants of the wild-type (*K*_d_ = 0.09 ± 0.01 nM) and the mCherry-conjugated ectodomain (*K*_d_ = 0.53 ± 0.19 nM) correspond well to the values found by others. Typically, the values range between 0.02 and 0.3 nM for full-length IGF-1R and between 0.3 and 0.9 nM for ectodomains in various studies [13,32,36].

**Table 1.** Binding characteristics of IGF-1R mutants. Wt means wild-type IGF-1R, and His697Ala and Arg704Ala are mutants of IGF-1R. nb – no binding. The program-generated value is artificial background noise.

mutation	whole IGF-1R transfected to R^−^ cells				isolated ecto-IGF-1R conjugated with mCherry			
	*B*_max_ ± S.D. [cpm]	% *B*_max_^a^	*K*_d_ ± S.D. [nM] (*n*)	% *K*_d_^b^	*B*_max_ ± S.D. [cpm]	% *B*_max_^a^	*K*_d_ ± S.D. [nM] (*n*)	% *K*_d_^b^
R^−^ cells	2157 ± 0225	10	nb (2)	nb				
Wt	21 329 ± 2025	100	0.09 ± 0.01 (3)	100	39 389 ± 4113	100	0.53 ± 0.19 (3)	100
His697Ala	11 087 ± 0666	52	0.38 ± 0.10 (3)	27	19 374 ± 1778	51	2.30 ± 0.33 (3)	23
Arg704Ala	16 974 ± 0611	80	0.34 ± 0.10 (3)	30	33 164 ± 2763	86	2.09 ± 0.64 (3)	25

^a^The maximum binding of the mutated receptor was compared to the maximum binding of the wild-type receptor and calculated as (*B*_max_ mutant/*B*_max_ wt) × 100 in each experiment separately (see electronic supplementary material, table S1), with the average value shown in the table.

^b^Relative equilibrium binding constant for IGF-1 of the mutant compared to the wild-type receptor was calculated as (*K*_d_ of wt/*K*_d_ of mutant) × 100 in each experiment separately (see electronic supplementary material, table S1), with the average value shown in the table. *n* indicates number of replicates.

The observed *K*_d_ values for the ectodomains compared to the whole receptors were higher (table 1), in accordance with known characteristics of IGF-1R [13]. Nevertheless, we found the same relative ratios among the data for ectodomains as for the whole receptors. The results were completely consistent between the two types of receptor constructs.

**Figure 3.**
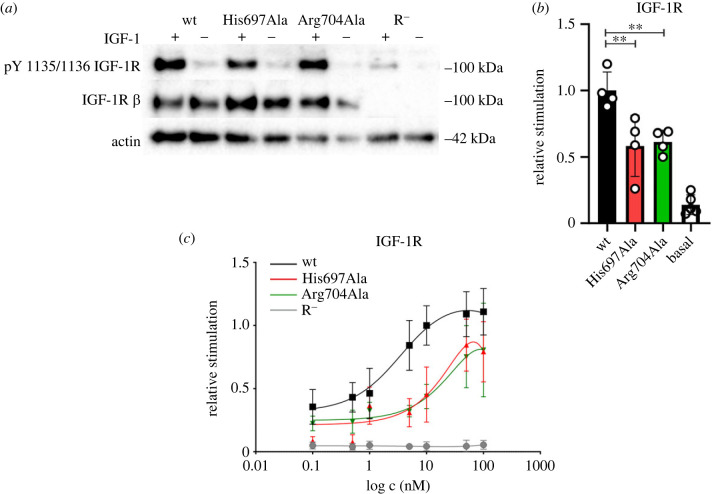
Stimulation of autophosphorylation of IGF-1R and its mutants by IGF-1. (*a*) Representative western blots. We present receptor expression and actin levels as a loading control and receptor autophosphorylation signal in stimulated (+) and non-stimulated (−) cells; stimulation was done by 10 nM IGF-1 for 10 min (for uncropped images, see electronic supplementary material, figure S1). (*b*) Quantitative comparison of autophosphorylation of the mutants relative to the wild-type IGF-1R (*n* = 4). The data were normalized to the receptor expression. Basal is the signal density in non-stimulated cells. Asterisks indicate where the activation of the receptor mutant by IGF-1 differs significantly from the wild-type hormone (**p < 0.01). (*c*) R^−^ cells transfected with the mutants on 96-well plates were stimulated with 0.1 nM to 100 nM of IGF-1 and receptor phosphorylation was measured using the In-cell Western assay (details are provided in the Methods section). The data with S.D. (*n* ≥ 3) are expressed as the contribution of phosphorylation relative to the signal of IGF-1 at 10 nM in wild-type IGF-1R-transfected cells.

The binding (in terms of the *K*_d_ value) of IGF-1 to the Arg704Ala mutant was decreased about four times compared to the wild-type receptor. This means that the binding of the mutant was compromised. The mutation of His697Ala resulted also in a four-fold decrease in binding (in terms of the *K*_d_ value), similar to the Arg704Ala mutant. Surprisingly, *B*_max_ of His697Ala was also decreased, namely by 50%. *B*_max_ should correspond to the amount of receptor binding sites available for binding. Considering that the concentration of the proteins was the same, as documented by Western blotting (figure 3; electronic supplementary material, figure S1) for full-length receptors and by fluorescence measurements for the ectodomains. This means that the number of binding sites available on the receptor was reduced to one-half.

Next, we tested the ability of the receptors transiently expressed in R^−^ cells to become auto-phosphorylated upon IGF-1 stimulation. Both mutants were stimulated with 10 nM IGF-1. The His697Ala mutant reached 58 ± 23% of the value of the wild-type receptor and the Arg704Ala mutant reached 61 ± 9% of the values of the wild-type receptor signal during the 10-min stimulation (figure 3*a,b*). We also measured the concentration-dependent stimulation curves (figure 3*c*) and showed that both mutants had a similar ability to become activated, reflecting their similar *K*_d_ values. The decreased *B*_max_ of the His697Ala mutant had no impact on the ability of the receptor to become auto-phosphorylated.

### 2.3. IR mutants

**Figure 4.**
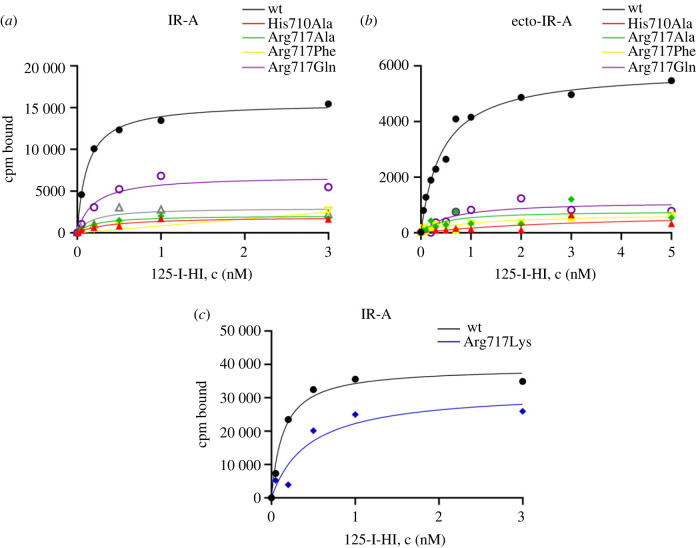
Representative saturation binding curves of IR-A mutants. (*a*) Whole receptors transiently expressed in R^−^ cells. (*b*) Isolated ectodomains attached to plates. (*c*) Arg717Lys mutant transiently expressed in R^−^ cells. ^125^I-HI is radioactively labelled human insulin. Individual curves including non-specific binding are presented in electronic supplementary material, figures S12–S13.

We performed the same set of experiments as for IGF-1R. Specifically, we mutated the IR-A *α*CT residues His710 and Arg717 to Ala. We prepared three more mutants of IR-A arginine, Arg717Gln, Arg717Phe and Arg717Lys. We performed saturation binding on R^−^ cells transfected with whole receptors. We also isolated the ectodomains conjugated with mCherry and performed saturation binding on plates. Representative binding curves are presented in figure 4. The individual binding curves including non-specific binding are shown in electronic supplementary material, figures S12–S13. The binding data are summarized in table 2 and complete data from all experiments are provided in electronic supplementary material, table S2.

**Table 2.** Binding characteristics of IR-A mutants. Wt means wild-type IR-A, and His710Ala, Arg717Ala, Arg717Phe, Arg717Gln and Arg717Lys are mutants of IR-A. nb – no binding. The program-generated value is artificial background noise.

mutation	whole IR-A transfected to R^−^ cells				isolated ecto-IR-A conjugated with mCherry			
	*B*_max_ ± S.D. [cpm] (*n*)	% *B*_max_^a^	*K*_d_ ± S.D. [nM] (*n*)	% *K*_d_^b^	*B*_max_ ± S.D. [cpm]	% *B*_max_^a^	*K*_d_ ± S.D. [nM] (*n*)	% *K*_d_^b^
R^−^ cells	1164 ± 354	7	nb (2)	nb				
Wt	12 693 ± 4.115 (5)	100	0.20 ± 0.08 (8)	100				
Wt^ c^	36 629 ± 2.263 (3)	100			5234 ± 462	100	0.61 ± 0.27 (3)	100
His710Ala	839 ± 510	9	nb (3)	nb	416 ± 322	7	nb (3)	nb
Arg717Ala	3391 ± 2869	24	0.79 ± 0.48 (5)	36	912 ± 128	18	1.23 ± 0.78 (3)	73
Arg717Phe	3462 ± 986	31	0.72 ± 0.21 (5)	42	685	12	1.10 (1)	39
Arg717Gln	4410 ± 1.836	36	0.27 ± 0.10 (5)	98	1259 ± 134	24	0.27 ± 0.10 (2)	95
Arg717Lys^c^	29 748 ± 3.532	82	0.55 ± 0.32 (3)	31				

^a^Maximum binding of the mutated receptor was compared to maximum binding of the wt receptor and calculated as (*B*_max_ mutant / *B*_max_ wt) × 100 in each experiment separately (see electronic supplementary material, table S2), with the average value shown in the table.

^b^Relative equilibrium binding constant for insulin of the mutant compared to the wt receptor was calculated as (*K*_d_ of wt / *K*_d_ of mutant) × 100 in each experiment separately (see electronic supplementary material, table S2), with the average value shown in the table; *n* indicates the number of replicates.

^c^The Arg717Lys mutant was compared to the wt receptor in a different set of experiments where transfection proceeded more effectively and the *B*_max_ was higher.

The measured wild-type IR-A (*K*_d_ = 0.2 ± 0.08 nM) corresponds well with the previously published data on high-affinity binding [11]. The *K*_d_ values determined in other works range between 0.09 and 0.5 nM [12,23,33]. However, binding to solubilized ectodomains gives *K*_d_ values of about 2–6 nM [37,38]. We determined *K*_d_ to equal 0.61 ± 0.27 nM. Our ectodomains were attached to plates and conjugated with the mCherry protein [39]. It is a monomeric variant of the tetrameric DsRed protein with improved characteristics. Nevertheless, we could see some aggregation of the isolated ecto-IR-mCherry conjugates (data not shown). Thus, conjugation with mCherry could place constraints on the ectodomains, resulting in a behaviour more similar to membrane-anchored insulin receptors [23]. Similarly to our results obtained with IGF-1R, the data were consistent between the two types of IR-A construcs.

The binding of ^125^I-TyrA14 human insulin to non-transfected R^−^ was negligible. The His710Ala mutant did not bind ^125^I-TyrA14 human insulin, in accordance with the critical role of this residue in insulin binding [31,34]. The binding of Arg717 mutants was also very low and not significantly increased above nonspecific binding for both the full-length receptors and the ectodomains (figure 4*a,b*; electronic supplementary material, figures S12–S13). Because of non-specific binding, the *K*_d_ values (table 2) should be interpreted with caution. Only the Arg717Gln mutant evidently showed some binding whereas the Arg717Ala and Arg717Phe mutants did not. We additionally prepared the Arg717Lys mutant to explore the importance of the positively charged residue in IR *α*CT 717 position. Indeed, this mutant was able to bind insulin effectively (figure 4*c*). However, the binding (in the terms of the *K*_d_ value) of Arg717Lys mutant reached only about 30% of the wild-type receptor. These data prove the critical role of the *α*CT Arg717 residue in ligand binding to IR-A.

**Figure 5.**
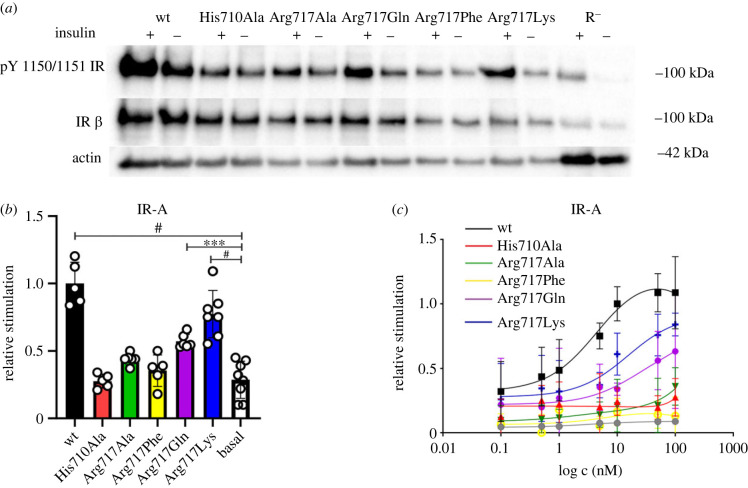
Stimulation of autophosphorylation of IR-A and its mutants by insulin. (*a*) Representative Western blots. We present receptor expression and actin levels as a loading control and receptor autophosphorylation signal in stimulated (+) and non-stimulated (−) cells; stimulation was done by 10 nM insulin for 10 min (for uncropped images, see electronic supplementary material, figure S2) (*b*) Quantitative comparison of autophosphorylation of the mutants relative to the wild-type IR-A (*n* ≥ 4). The data were normalized to the receptor expression. Basal is the signal density in non-stimulated cells. Asterisks indicate where the activation of the receptor mutant by insulin differs significantly from the basal value (***p < 0.001; ^#^*p* < 0.0001). (*c*) R^−^ cells transfected with the mutants on 96-well plates were stimulated with 0.1 nM to 100 nM insulin, and receptor phosphorylation was measured using the In-cell Western assay (details are provided in the Methods section). The data with S.D. (*n* ≥ 3) are expressed as the contribution of phosphorylation relative to the signal of insulin at 10 nM in wild-type IR-A-transfected cells.

We also performed signalling experiments. These experiments were hampered by high background signal. The transfected receptors in cells appeared to be autophosphorylated even without stimulation by insulin. We think that it was due to the transient character of the transfection (figure 5). Nevertheless, data from multiple signalling experiments, after subtraction of the background, followed the results of the binding experiments. The wild-type IR-A and the Arg717Lys mutant induced receptor autophosphorylation. The His710Ala, Arg717Ala and Arg717Phe mutants were not activated by insulin over the background level whereas the Arg717Gln mutant showed activation significantly greater than the background (figure 5*b*).

## 3. Discussion

Tremendous progress in our understanding of how insulin and IGF-1 bind to their receptors has been made since the first structure of the complex was solved [34]. This is mainly due to great advances in cryo-EM technology. Although various conformational states of the receptor–ligand complexes have been revealed, the mechanism of the transition from the apo-form to the signalling conformation is not yet fully understood [16]. In this respect, our mutational analysis provides some interesting new data.

To characterize our mutants, we performed saturation binding experiments. We had no ambition to investigate the binding kinetics in detail. This can be done by performing competitive binding assays and measurements of ligand dissociation rates using picomolar concentrations of a labelled tracer [11–13,37]. We performed our saturation binding experiments with an excess of the ligand (0.05–3 nM for the full-length receptor and 0.05–5 nM for the ectodomains). Thus, we neglected the steps comprising the high-affinity and low-affinity interactions and structure reorganization events and considered only the transition between an unbound and a fully saturated state. Although we were aware that the conformation of insulin molecules at Site 1 (detached B-chain) differs from that at Site 2 [5] and that the potential Site 2 of IGF-1R is unknown [25], the binding of multiple ligands to their binding sites was considered as a single step and one-site curve fitting was applied. The observed *B*_max_ would then correspond to four binding sites on the insulin receptor, as it was shown in saturated structures [5,20] (figure 1*b*) and two binding sites on IGF-1R, as follows from the principle of negative cooperativity [14]. Our binding constants (*K*_d_) correspond well to those computed for high-affinity complexes [11]. Despite the simplified approach, the presented saturation curves allowed us to reveal the global impact of the mutations on the receptor binding. (i) We have reproduced the data obtained by others and shown that mutation His710Ala of the insulin receptor inactivates it [31,33]. (ii) By contrast to the previous presumptions [31], we found that the mutation Arg717 of the insulin receptor has a damaging effect on the binding of insulin to its receptor and highlights the importance of a positively charged residue in *α*CT position 717. (iii) We have proved the involvement of the IGF-1R *α*CT residue Arg704 in IGF-1 binding to the IGF-1R. (iv) We have confirmed the important role of the IGF-1R *α*CT residue His697 for IGF-1 binding to the IGF-1R, known from previous studies [32,40]. Moreover, we have shown that the number of binding sites for IGF-1 on IGF-1R is reduced to one-half in the His697 mutant compared to the wild-type receptor. The impact of mutations of the *α*CT residues on the receptors differs between the insulin receptor and IGF-1R.

### 3.1. Mutation of Arg704 in IGF-1R

**Figure 6.**
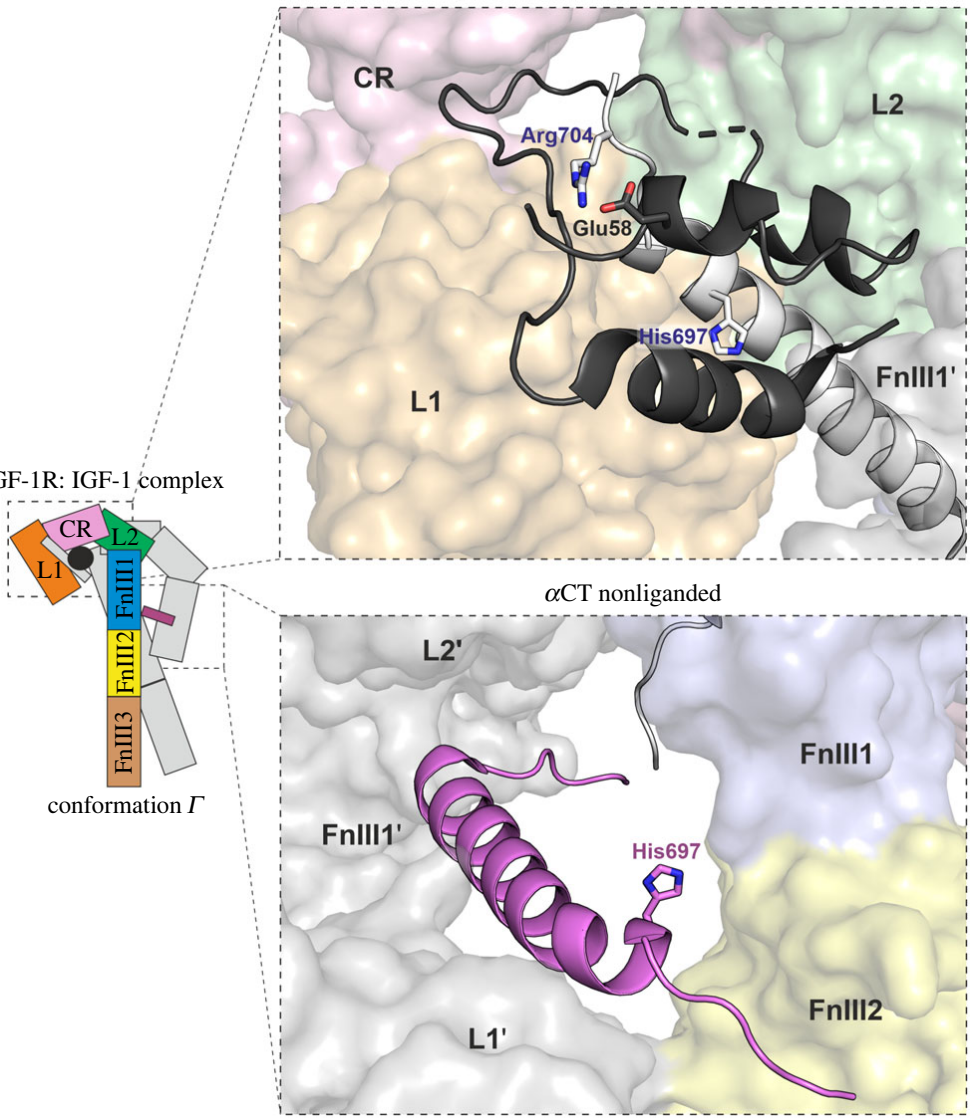
Close-up views of the structure of IGF-1 bound to IGF-1R (PDB ID: 6PYH). The schematic organization of the domains is the same as in figure 1 of this manuscript. Receptor domains are named and colour-coded on one half of the dimer. The second part is sketched. The colour scheme is the same in the close-up views. The receptor is shown as a surface, and the *α*CTs and IGF-1 are shown as ribbons. The locations of IGF-1R Arg704 and His697 are highlighted in both *α*CTs and depicted as sticks. The position of IGF-1 Glu58 is also shown. Arg704 was not resolved in the unliganded *α*CT.

Our study was initially inspired by our mutational survey of suggested Site 2 residues in insulin and their analogous positions in IGF-1 and IGF-2 [28,30]. Our IGF-1 Glu58His and Glu58Asp analogues were markedly less potent than native IGF-1. Based on the crystal structure published by Xu *et al.* [21], we proposed that IGF-1 Glu58 might interact with the IGF-1R *α*CT residue Arg704. No mutational study of the IGF-1R Arg704 residue had been carried out, so we undertook this task ourselves. The results indicate that the mutation of Arg704 indeed affects the binding of IGF-1 to the receptor through an increase in the equilibrium binding constant *K*_d_. This means that the association of the ligand with the receptor is slowed down, or that the dissociation from the complex is faster, or that both processes are impaired to a certain extent [41]. Close-up views of IGF-1 bound to IGF-1R are presented in figure 6 illustrating the close contacts of Glu58 with Arg704. Interestingly, in the structure of IGF-2 bound to the IGF-1R ectodomain stabilized with a leucine-zipper motif [36], Arg704 was shown to interact with IGF-2 Thr58 and Thr62 instead of Glu57. This explains our previous observation that the mutation of IGF-2 Asp57 had no impact on receptor binding [28].

### 3.2. Mutation of His697 in IGF-1R

Rather surprising conclusions were reached for the mutation of IGF-1R His697. This residue was mutated in two previous studies [32,40], both of which obtained similar results to ours. We found a four-fold increase in the *K*_d_ of IGF-1 binding to the mutated receptor, compared to the wild-type receptor. Mynarcik *et al*. [40] reported a similar, 5.6-fold increase. However, we have also observed a reduction of the maximum binding of the His697Ala mutant by 50%. This was not appreciated in previous works, because they estimated the *K*_d_ based on results of competitive binding experiments, where changes in *B*_max_ are not immediately obvious. The simplest explanation of the lowering of *B*_max_ is that the number of binding sites on the receptor is decreased to one-half. A similar result was obtained by Uchikawa *et al*. [20], who mutated Site 2 residues of the insulin receptor and observed a decrease in maximum binding to approximately one-half. However, four insulins were shown to be attached to the insulin receptor and a mutation of the Site 2 position resulted in the binding of just two insulins to Site 1. As concerns IGF-1R, only a single ligand was found to be in complex with IGF-1R even under excess of the ligand [24,42]. The conclusion was that two ligands cannot be simultaneously bound to the active conformation of IGF-1R. Recently, papers describing the structures of IGF-1R in complex with two ligands have appeared. These structures seem to represent inactive forms of IGF-1R. Moreau *et al*. [25] solved the structure of IGF-1R in complex with an IGF-1R antagonist. The complex displayed a pseudo-two-fold symmetric arrangement with two ligands bound. The FnIII-3 domains remained separated in this conformation and thus prevented the activation of the receptor kinase. Another paper by Li *et al*. [26] described the structure of an IGF-1R mutant with a disrupted tether coupling the two *α*CTs in the IGF-1-bound state. They found a minor class of particles with a symmetrical T-shape conformation with two IGF-1 molecules bound. They observed an increased maximum binding of these mutant receptors but lower receptor autophosphorylation, concluding that the optimal active conformation of the receptor was disrupted by the second ligand. Also, Wu *et al*. [27] presented a map with two IGF-1 bound, the position of which differed from that of IGF-1 bound to IGF-1R in active conformation, and the FnIII-3 domains seem to be separated.

Close-up views of IGF-1 bound to IGF-1R are presented in figure 6. His697 interacts with the B-domain helix (Thr4–Arg21) of IGF-1. Upon inspection of the structure (PDB ID: 6PYH) [7], we can see the His697 residue of the unliganded *α*CT ‘sitting’ at the edge of a rigidly complexed *α*CT bound to the unliganded L1 domain (figure 6). This His697 residue is probably involved in the binding of a second IGF-1 molecule as it makes contact with the receptor. The mutation of His697 might prevent such an interaction, resulting in a reduction of potential binding sites to one-half. The IGF-1:IGF-1R complex can probably be loosened through the binding of a second IGF-1 molecule to this exposed His697 residue, and the first bound IGF-1 is then released in agreement with the mechanism of negative cooperativity. The conformation of double-liganded IGF-1R yields a partially inactivated receptor and might be highly unstable, but our data, together with the negative cooperativity property of IGF-1R, strongly suggest that double-liganded IGF-1R may indeed exist, albeit only for a short time.

### 3.3. Mutation of His710 in the insulin receptor

We mutated the His710 residue to show that we can inactivate the receptor and to prove that our methods work. We wanted to have a negative control because we did not expect the mutation of the Arg717 to have any impact on receptor activity, but the data showed otherwise.

### 3.4. Mutation of Arg717 in the insulin receptor

The IR Arg717 position has never been highlighted as being important for insulin binding [20,23]. The only mutational analysis of this residue, performed by Mynarcik *et al*. [31], labelled it unimportant. Quite unexpectedly, the Arg717 mutation had a damaging effect on IR-A binding. Although our data on IR-A were limited by non-specific binding and a high background in both binding and autophosphorylation experiments, the results obtained with Arg717 mutants were similar to those obtained with the inactive His710Ala mutant. We additionally prepared the Arg717Lys mutant to prove that the binding of insulin to our receptor construct could be restored. We prepared four IR Arg717 mutants with different side-chain length and characteristics: an Ala mutant with a short and inert side-chain, a Phe mutant with a long and aromatic side-chain, a Gln mutant with a long and polar side-chain containing the amide group but not preserving the basic character of Arg, and an Arg717Lys mutant that preserved the basic character of the side chain. This mutant bound insulin effectively but less strongly than the wild-type insulin receptor, confirming the importance of the strongly basic guanidino group. Among the other mutants, only the Arg717Gln mutant repeatedly provided some measurable data (table 2; figure 4; electronic supplementary material, table S2) and was also autophosphorylated after insulin stimulation (figure 5). This evokes the idea that the interaction of insulin with the Gln amide at position 717 could trigger a partial movement of the insulin receptor domains but that the receptor is not allowed to effectively transition into a high-affinity complex capable of binding other insulin molecules.

**Figure 7.**
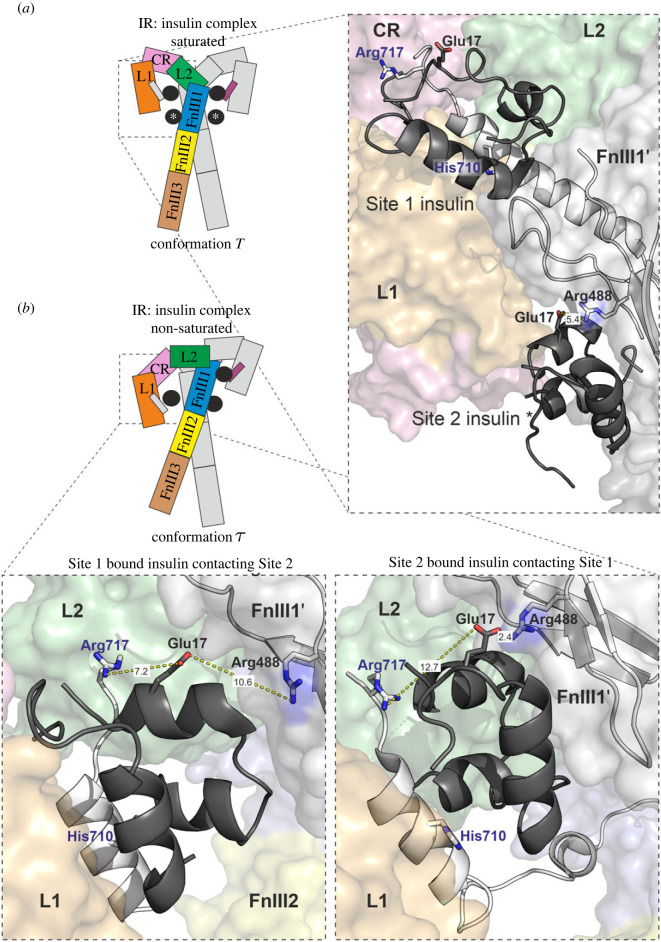
Close-up views of the structure of insulin bound to the insulin receptor. (*a*) Insulin-saturated conformation (PDB ID:6SOF) showing insulin at Site 1 and at Site 2. (*b*) Insulin-nonsaturated structure of three insulin molecules bound to the insulin receptor. Close-up views show two different positions of insulins in combined Sites 1–2 (PDB ID: 7STJ on the left and PDB ID: 7STK on the right). The schematic organization of the domains is the same as in figure 1 of this manuscript. Receptor domains are named and colour-coded on one half of the dimer; the second part is sketched. The colour scheme is the same in the close-up views. The receptor is shown as surface and the *α*CTs and insulins are shown as ribbons. The locations of insulin GluA17 and insulin receptor Arg717 and His710 are highlighted and depicted as sticks. Distances (in Å) of GluA17 from Arg717 in *α*CT and Arg488 in FnIII-1 were measured and are indicated as yellow dotted lines.

We tried to explain why Arg717 is so important for insulin binding by inspecting published structures. Li *et al*. [6] published two asymmetric insulin receptor conformations, showing combined Site 1–Site 2-bound insulin, where insulin attached to Site 2 weakly interacts with Site 1 (PDB ID: 7STK), and *vice versa*, insulin attached to Site 1 interacts with Site 2 (PDB ID: 7STJ). We show in figure 7 the combined Sites 1 and 2 and also the positions of insulins at Site 1 and Site 2 in a fully saturated insulin receptor complex (PDB ID: 6SOF). It has been suggested by others that Site 2 is the site of the first contact [22,23,37]. GluA17 was shown to interact with the FnIII-1 domain Arg488 as part of Site 2 on insulin [5] (figure 7*a*). GluA17 seems to be rotated away from the FnIII-1 domain when insulin approaches Site 1 (PDB ID: 7STJ; yellow dotted lines in figure 7). IR *α*CT Arg717 interacts also with insulin AsnA21 when attached to the combined Site 1–2 (PDB ID: 7STK). Neither Arg717 nor GluA17 and AsnA21 are embraced in the insulin Site 1-bound structure (PDB ID: 6SOF; figure 7*a*). However, all these residues were shown to be important for insulin binding. The insulin mutation of AsnA21 and that of GluA17 resulted in reduced binding to the IR ectodomain [28,43]. Our mutation of Arg717 resulted in an almost inactive receptor. We speculate that insulin attached to the Site 2 on the receptor is trapped by the C-terminus of *α*CT during dynamic fluctuations of the receptor domains, which involves an ionic interaction mediated by Arg717. The insulin is then rolled over the receptor domains to its Site 1-bound position. Our study demonstrates the important role of IR Arg717 in IR binding and suggests this residue, together with GluA17 and AsnA21, is involved in the transition between the apo-form and the insulin-bound state. This result was not predicted based on available structures of IR–insulin complexes.

## 4. Methods

### 4.1. Transfection plasmids

The full-length clone of human IR-A and human IGF-1R in a pCMV3 vector with a C-terminal HisTag were purchased from Sino Biological (IR cat. no. HG11086-CH and IGF-1R cat. no. HG10164-CH). Oligonucleotides were ordered from Generi Biotech or Sigma-Aldrich. The Phusion Polymerase, T4 ligase and restriction enzyme kits (HindIII, BamHI and DpnI) were obtained from ThermoFisher. Plasmids were purified using the ZymoPure Plasmid MiniPrep kit (Zymo Research) or the NucleoBond Xtra Midi kit (Macherey Nagel). The NucleoSpin Gel and Clean-up kit (Macherey Nagel) were used to purify PCR products from gel.

Mutations Arg717Ala, Arg717Phe, Arg717Gln, Arg717Lys and His710Ala in IR and Arg704Ala and His697Ala in IGF-1R were introduced into the sequences by site-directed mutagenesis (primer sequences are shown in electronic supplementary material, table S3). The PCR reaction with forward and reverse complementary primers bearing the desired mutation was performed and the template vector was destroyed by DpnI. Competent TOP10 cells were transformed with the PCR product and grown on agar plates with selection antibiotics. Plasmids from several colonies were isolated and sequenced (Eurofins Scientific). Clones with a confirmed mutation were propagated further.

Ectodomains (residues 1–944 IR and 1-932 IGF-1R) of wild-type (wt) and mutated receptors were cloned into a modified pTT5SH8Q2 vector (NRC Biotechnology Research Institute, Canada), using HindIII and BSp119I restriction sites. The vector modification consisted of the introduction of the mCherry protein gene to be attached C-terminally with a linker sequence (FEGGSGGVD) containing suitable restriction sites. Plasmids were propagated in TOP10 competent cells.

### 4.2. Cell line transfection with full-length receptors

Full-length receptors and their mutants (IR Arg717Ala, Arg717Phe, Arg717Gln, Arg717Lys, His710Ala, IGF-1R, Arg704Ala, His697Ala) were transiently expressed in mouse embryonic fibroblasts, derived from animals with the targeted disruption of the IGF-1 receptor gene (R^−^ cells, kindly provided by Professor R. Baserga, Thomas Jefferson University, Philadelphia, PA, USA) [44]. The R^−^ cells were grown in DMEM with 5 mM of glucose (Biosera) supplemented with 10% fetal bovine serum and 2 mM of L-glutamine, in humidified air with 5% CO_2_ at 37°C. The cells were seeded in 24-well plates (2 × 10^4^ cells per well). After 24 h, the cells in the respective wells were transiently transfected with full-length human IR, IGF-1R or mutants (250 ng DNA per well) using the Lipofectamine 2000 reagent (ThermoFisher). All receptors were expressed with ten His residues at their C terminus. The effectivity of the transfection in each experiment was checked by Western Blotting, using an anti-IR β-subunit antibody (Invitrogen, cat. no. AHR0271) or an anti- IGF-1R *β* (111A9) antibody (Cell Signaling, cat. no. 3018). An anti-actin (20–33) antibody (Sigma-Aldrich, cat. no. A5060) was used as a loading control.

### 4.3. Cell line transfection with receptor ectodomains and ectodomain isolation

The ectodomains (residues 1–944 IR and 1–932 IGF-1R) of the receptors and their mutants were expressed in the Hek293-6E clone of the human embryonic kidney cell line (NRC Biotechnology Research Institute, Canada). The ectodomains were produced as fusion proteins with the C-terminally linked mCherry fluorescent protein (pmCherry-N1) and HisTag consisting of eight His residues. The Hek293-6E cells were grown in F17 medium (Invitrogen) supplemented with 0.1% Kolliphor and 4 mM of L-glutamine in humidified air with 5% CO_2_ at 37°C. They were grown in Erlenmeyer flasks and constantly agitated at 120 rpm. Cells in the density 1.5 × 10^6^ ml^−1^ were transfected with linear polyethyleneimine (PEI) (Polysciences). The DNA was mixed with PEI (1 mg ml^−1^) at a ratio of 1 : 4; 1 g of DNA was used per 10^6^ cells. The cells were grown for five days. Cells were removed by spinning at 1200 rpm for 5 min and the supernatants were centrifuged for another 5 min at 4000 rpm. The ectodomains were isolated from the supernatants by affinity chromatography on His-Select Nickel Affinity Gel (Sigma-Aldrich), according to the manufacturer's standard procedure. The fraction after elution was desalted by dialysis in PBS. The concentration of the ectodomains was estimated by measuring fluorescence at 587 nm. The effectivity of the transfection was checked by Western Blotting, using an anti-IR α-subunit antibody (Invitrogen, cat. no. AHR0221) or an anti-IGF-1R alpha (24–60) antibody (ThermoFisher, cat. no. MA5-13817). The mutations were confirmed using mass spectrometry analysis.

### 4.4. Mass spectrometry

Isolated receptor ectodomains were loaded onto SDS polyacrylamide gels, and separated α-subunits were digested with trypsin and analysed using the Vanquish liquid chromatography system (ThermoScientific) connected to a timsToF SCP mass spectrometer equipped with a Captive Spray source (Bruker Daltonics). The mass spectrometer was operated in a positive data-dependent mode. One microlitre of the peptide mixture was injected by an autosampler into the C18 trap column (PepMap Neo C18, 5 µm, 300 µm × 5 mm, Thermo Scientific). After 3 min of trapping, peptides were eluted from the trap column and separated in the C18 column (DNV PepMap Neo 75 µm × 150 mm, 2 µm, Thermo Scientific) by a linear 35-min water−acetonitrile gradient from 5% (v/v) to 35% (v/v) acetonitrile at a flow rate of 350 nl min^−1^. The trap and analytical columns were both heated to 50°C. Parameters from the standard proteomics PASEF method were used to set timsTOF SCP. The scan range was set between 0.6 and 1.6 V  s cm^−2^ with a ramp time of 100 ms. The number of PASEF MS/MS scans was 10. Precursor ions in the m/z range between 100 and 1700 with charge states of ≥ 2+ and ≤ 6+ were selected for fragmentation. Active exclusion was enabled for 0.4 min. The data were searched for IR-A or IGF-1R with the introduction of expected mutations in their sequences. Carbamidomethylation of cysteine and possible single oxidation of methionine were considered stable and variable modifications, respectively, in the searches.

### 4.5. Binding experiments on cells, full-length receptors

Cells 24 h post-transfection were washed with binding buffer (100 mM HEPES/NaOH, pH 7.6, 100 mM NaCl, 5 mM KCl, 1.3 mM MgSO_4_, 1 mM EDTA, 10 mM glucose, 15 mM sodium acetate and 1% bovine serum albumin). The cells were incubated for 16 h at 5°C with increasing concentration of ^125^I-TyrA14 human insulin [45] (the specific activity of ^125^I-TyrA14 insulin is 2200 Ci  mmol^–1^) for saturation binding to the insulin receptor and insulin receptor mutants. The cells were incubated with ^125^I-IGF-1 (prepared in house; for the method of its preparation and characterization, see electronic supplementary material, Methods; the specific activity of ^125^I-IGF-1 is 2100 Ci  mmol^–1^) for saturation binding to IGF-1R and IGF-1R mutants. After incubation, the cells were washed twice with ice-cold binding buffer and solubilized with 0.1 M NaOH. The cell-associated radioactivity was measured using the 2470 WIZARD2 Automatic Gamma Counter (Perkin Elmer). Nonspecific binding was measured with an excess of unlabelled ligand 1 µM insulin or 1 µM IGF-1 for each radioligand concentration and subtracted from the saturation curve. The data were analysed in GraphPad Prism 8, using a nonlinear one-site specific binding fitting program. The measurements were repeated at least three times. The binding of control non-transfected R^−^ cells was negligible. We repeated the experiment only twice to preserve the radioactive ligands.

### 4.6. Binding experiments on plates, receptor ectodomains

Saturation binding to IR and IGF-1R ectodomains and their mutants was performed by adapting the method developed by Potalitsyn [46], using a modular polymer-based synthetic antibody (iBody, IOCB Tech). Briefly, receptor ectodomains (300 ng well^–1^ as estimated based on mCherry fluorescence) were immobilized through their HisTag on 96-well plates by nickel-charged NTA (tris-nitrolacetic acid)-decorated iBodies containing biotin [47] attached to a plate pre-sorbed with neutravidin. The saturation experiments were performed essentially in the same way as described for the whole-cell assay and in Potalitsyn [46]. The measurements were repeated three times. However, the binding on the ectodomains of mutated receptors was negligible and only one or two measurements were performed for the IR Arg717Phe and IR Arg717Gln mutants to preserve the radioactive ligand.

### 4.7. Signalling

Stimulation of the autophosphorylation of receptors in the transiently transfected R^−^ cells was performed 48 h post transfection, as described in Krizkova [48]. The cells were stimulated for 10 min with 10 nM human insulin or 10 nM IGF-1. The phosphorylation of the receptors was analysed by Western Blots with an anti-Phospho-IGF-1 Receptor *β* (Tyr1135/1136)/Insulin Receptor *β* (Tyr1150/1151) (19H7) antibody (Cell Signaling, cat. no. 3024). The quantity of the transfected receptors was determined using an anti-IR β-subunit antibody (Invitrogen, cat. no. AHR0271) or an anti-IGF-1R *β* (111A9) antibody (Cell Signaling, cat. no. 3018). The phosphorylation signal was normalized to the whole receptor expression. The experiment was repeated at least four times. The significance of differences in autophosphorylation ability of the receptor mutants and wild-type receptors was calculated, using one-way analysis of variance with Dunnett's test comparing the autophosphorylation of mutated receptors with the background signal of non-stimulated cells or with the wild-type receptor. An anti-actin (20–33) antibody (Sigma-Aldrich, cat. no. A5060) was used as another loading control.

Ligand–dose response receptor autophosphorylation levels were determined using an In-Cell Western assay adapted for chemiluminescence as described in Macháčková *et al*. [49]. The transient transfection step was included in the assay. Briefly, the R^−^ cells were plated at 5000 cells/well on white 96-well cell-grade Brand plates (Brand GMBH, Germany) and incubated for 24 h. The cells were transfected with the respective full-length wild-type and mutant receptor plasmids and incubated for another 24 h. Next, the transfected cells were starved for 4 h in serum-free media and stimulated with dilutions of ligands for 20 min. After incubation, the cells were fixed in 3.75% freshly prepared formaldehyde for 20 min. The cells were permeabilized with 0.1% Triton-X-100 in PBS for 5 min and blocked with 5% BSA in T-TBS. The plates were incubated with anti-phospho-IGF-1R*β* (Tyr1135/1136) / IR*β* (Tyr1150/1151) overnight at 4°C and developed with a peroxidase-labelled anti-rabbit secondary antibody (Sigma). Control wells were incubated with an anti-IR β-subunit antibody or an anti-IGF-1R *β* (111A9) antibody to estimate the level of transfection. SuperSignal The West Femto maximum sensitivity substrate was added to each well, and chemiluminescence was detected using the ChemiDoc MP Imaging System. The data were subtracted from the background values and expressed as the contribution of phosphorylation relative to 10 nM insulin or to the 10 nM IGF-1 signal of wild-type receptors. Each point was measured in duplicate, and the experiment was repeated twice. Control non-stimulated wells for each mutant (background) and wells for whole receptor expression were conducted as tetraplicates on each plate. Nonlinear regression curve fitting of the combined data from all experiments (i.e. 4 values per point) was carried out using GraphPad Prism 5 software.

## Data Availability

Representative raw data (Western blots, binding curves and MS/MS data) are provided in the electronic supplementary material [50].
